# Heroes in endocrinology: Nobel Prizes

**DOI:** 10.1530/EC-14-0070

**Published:** 2014-08-28

**Authors:** Wouter W de Herder

**Affiliations:** 1 Section of Endocrinology, Department of Internal Medicine Erasmus MC 's Gravendijkwal 230, Rotterdam, 3015 CE The Netherlands

**Keywords:** diabetes, pituitary, thyroid, adrenal, neuroendocrinology

## Abstract

The Nobel Prize in Physiology or Medicine was first awarded in 1901. Since then, the Nobel Prizes in Physiology or Medicine, Chemistry and Physics have been awarded to at least 33 distinguished researchers who were directly or indirectly involved in research into the field of endocrinology. This paper reflects on the life histories, careers and achievements of 11 of them: Frederick G Banting, Roger Guillemin, Philip S Hench, Bernardo A Houssay, Edward C Kendall, E Theodor Kocher, John J R Macleod, Tadeus Reichstein, Andrew V Schally, Earl W Sutherland, Jr and Rosalyn Yalow. All were eminent scientists, distinguished lecturers and winners of many prizes and awards.

## Introduction

Among all the prizes awarded for life achievements in medical research, the Nobel Prize in Physiology or Medicine is considered the most prestigious.

The Swedish chemist and engineer, Alfred Bernhard Nobel (1833–1896), is well known as the inventor of dynamite and the owner of the company Bofors, which manufactured armaments. Disappointed by the public image of him as ‘the merchant of death’, Nobel sought to alter the negative perception of his legacy by leaving his fortune to be used posthumously for the establishment of the Nobel Prize Trust. The award of prizes for pre-eminence in five individual fields: physical science, chemistry, medicine or physiology, literature and peace. Given his penchant for the development of inventions related to war and death, it is paradoxical that Alfred Nobel had also an active interest in medical research. In fact, the award for Physiology or Medicine was the third prize defining his will of 1895 where he proposed the establishment of the concept of recognising global merit.

The Nobel Prize in Physiology or Medicine is awarded annually by the 50 voting members of the Nobel Assembly at the Karolinska Institutet in Stockholm (Sweden) (1, 2). In 1901, the first prize was awarded to the German physiologist Emil A von Behring (3, 4). This award heralded the first recognition of extraordinary advances in medicine that has become the legacy of Nobel's prescient idea to recognise global excellence. It is noteworthy that the First Nobel Prize in Physics of the same year was awarded to Wilhelm C Röntgen for the discovery of X-rays. This advance presaged the subsequent application to the field of medicine and laid the basis for the development of the interdisciplinary application of science.

In the years that have elapsed since the initiation of the concept, the Nobel Prize has also been awarded to several distinguished endocrinologists. Their achievements and lives are briefly reviewed in this text, and summarised in Table 1.

## The Nobel Prize in Physiology or Medicine 1909

The Nobel Prize in Physiology or Medicine 1909 was awarded to Emil Theodor Kocher ‘for his work on the physiology, pathology and surgery of the thyroid gland’ (5).

### Theodor Kocher

Emil Theodor Kocher was born on 25 August 1841 in Bern (Switzerland) and schooled in Burgdorf (Switzerland) and Bern. He undertook his doctorate studies in Bern under the leadership of Michael Anton Biermer and in 1865 obtained his Doctorate ‘summa cum laude unanimiter’. Thereafter, Kocher joined the staff of the German surgeon C A Theodor Billroth who was at that time Professor and Director of the University Surgical Hospital and Clinic in Zurich (Switzerland). Kocher then travelled Europe and consorted with many of the famous surgeons of his time. From 1865 to 1867, he worked with Bernhard R K von Langenbeck, Director of the Clinical Institute for Surgery and Ophthalmology at the Charité, Berlin (Germany), before in 1867 visiting London (UK) to undertake further study with Sir Henry Thompson, Professor of Clinical Surgery, and Sir John Eric Erichsen, Professor of Surgery, at the University College Hospital. In 1867, he also travelled to Paris to meet with the surgeon Auguste Nélaton, the chemist Auguste V L Verneuil and the chemist and microbiologist Louis Pasteur. His (sponsored) travels gave him exposure to diverse scientific concepts and also enabled him to acquire and develop many novel surgical techniques. In 1867, he returned to Bern where he prepared for his ‘habilitation’ and was granted the ‘venia docendi’. At this time, he was appointed assistant to Georg A Lücke, whom he succeeded in 1872 as Ordinary Professor of Surgery and Director of the University Surgical Clinic at the Inselspital Bern. In 1869, he married Marie Witschi-Courant (1841–1921) and the couple had three children.

In his lifetime, Kocher established himself as a legendary teacher and clinician authoring almost 250 medical papers and textbooks while training an entire generation of surgeons. He served in 1900 as the mentor of Harvey W Cushing, the founder of neurosurgery and the pioneer of the evolution of pituitary surgery and pituitary disease. Cushing worked for several months in the laboratory of Kocher and addressed the problem of the regulation of cerebral vascular perfusion (6). Kocher achieved pre-eminence for his advances in endocrine surgery, especially by reducing the mortality of thyroidectomies from as high as 75% to below 1%!! So effective was his surgical resection of goitre, however, that the complete extirpation of all thyroid tissue carried its own consequences. Thus, in 1882, the Swiss cousins and surgeons Jacques-Louis and Auguste Reverdin first reported that myxoedema occurred as a delayed complication of total thyroidectomy (7, 8, 9, 10, 11, 12). This adverse effect was unmanageable until 1953 when thyroid hormone replacement therapy became available (13, 14, 15). As a consequence of these observations, Kocher also came to the conclusion that a complete removal of the thyroid was not to be recommended and reported this to the German Surgical Society in 1883 (11, 16, 17, 18, 19). Nine years after a total thyroidectomy in a young female, Kocher had noted very substantial changes as compared with her younger sister, who in the past had closely resembled her. He reported: ‘whilst the younger sister has now grown up to a blossoming young woman of very pretty looks, the sister operated on has remained small and exhibits the ugly looks of a semi-idiot’. Kocher immediately reviewed all goitre patients he had operated on and noted considerable differences between those in whom he had undertaken a partial thyroidectomy and those in whom he had performed a total thyroidectomy. While partially thyroidectomised patients were generally in good health and ‘very happy with and grateful for the success of the operation’, only two of the total thyroidectomised patients showed improvements.

Kocher was an innovative administrator as well as surgeon and was responsible for the modernisation of the Inselspital 1884–1885 and Rector of the University in 1878 and in 1903. In addition to these University activities from 1905, he owned a small private clinic called ‘Ulmenhof’ where he treated many wealthy and famous patients.

On the evening of 23 July 1917, Theodor Kocher performed his last emergency operation. Feeling unwell thereafter, he retired to his bed where he lost consciousness and died 4 days later on 27 July 1917, aged 75 years (20).

## The Nobel Prize in Physiology or Medicine 1923

The Nobel Prize in Physiology or Medicine 1923 was awarded to Frederick Grant Banting and John James Rickard Macleod ‘for the discovery of insulin’.

### Frederick Banting

Sir Frederick Grant Banting was born on 14 November 1891 near Alliston, Ontario (Canada), and subsequently studied medicine at the University of Toronto where he received a Bachelor of Medicine Degree in 1916. As a physician, he enlisted in the Canadian Army Medical Corps and in 1918 was wounded at the battle of Cambrai (France). His heroism was recognised by the award of the Military Cross in 1919. At the cessation of the war, Banting returned to Canada and initially entered general practice in London, Ontario, before in 1919 undertaking an orthopaedic residency at the Hospital for Sick Children in Toronto for a year. From 1921 to 1922, he lectured in pharmacology at the University of Toronto, receiving the gold medal with the award of his MD in 1922.

In the beginning of the 20th century, several distinguished scientists including the German pathologist Bernhard Naunyn, the Lithuanian internist Oskar Minkowski, the English physiologist Sir Edward A Sharpey-Schafer and the American pathologist Eugene L Opie all addressed issues of metabolism, especially glucose homoeostasis. In this respect, the focus was the identification and isolation of a pancreatic agent, later named ‘insulin’ and its involvement in the regulation of blood glucose levels. A critical experimental problem was the difficulty of extracting insulin from the pancreas before its enzymatic degradation. In 1920, Frederick Banting approached John J R Macleod, Professor of Physiology at the University of Toronto, and suggested to him an approach for the isolation of insulin from the pancreas. Macleod provided him laboratory space, experimental animals and the assistance of one of his students, Charles H Best, who worked as a demonstrator. Banting and Best isolated insulin from the pancreas and successfully reduced the blood glucose levels in a diabetic dog, whose pancreas had been surgically removed (21, 22). In 1922, Banting was appointed Senior Demonstrator in Medicine at the University of Toronto and the following year he was elected to the new Banting and Best Chair of Medical Research. He also served as Honorary Consulting Physician to the Toronto General Hospital, the Hospital for Sick Children in Toronto and the Toronto Western Hospital.

In 1924, Banting married Marion Robertson (1896–1944), but the marriage was dissolved in 1932. In 1934, he was knighted by King George V and in 1937 he married Henrietta Ball (1912–1976).

His subsequent research (1938) in conjunction with the Royal Canadian Air Force (RCAF) involved the physiological problems (syncope) encountered by pilots flying high-altitude fighter planes. In February of 1941, Frederick Banting perished at the age of 49, en route to England when his plane crashed in Musgrave Harbour, Newfoundland (Canada). In 2004, Frederick Banting, having discovered insulin and the recipient of numerous honours and much acclaim, was voted into fourth place as The Greatest Canadian (22).

### John Macleod

John James Rickard Macleod was born on 6 September 1876 in Clunie, Scotland, and studied medicine at the University of Aberdeen. In 1898, he received a PhD in Medicine and thereafter studied biochemistry at the University of Leipzig (Germany) for a year, before becoming a demonstrator at the London Hospital Medical School in 1900 and in 1902 Lecturer in Biochemistry. In the same year, he was awarded a Doctorate in Public Health from Cambridge University. In 1903, he married Mary Watson McWalter, his second cousin and immigrated to the United States having accepted a position as a lecturer in physiology at the Case Western Reserve University in Cleveland, Ohio. During his initial years at Case Western Reserve University, Macleod indicated an interest in carbohydrate metabolism and this focus would more than a decade later be rekindled in his association with Frederick Banting. At the cessation of the First World War (1918), he became Director of the Physiology Laboratory and Assistant to the Dean of the Medical Faculty at the University of Toronto (Canada). In 1920, Macleod, Banting and Best began collaborating on the issue of glucose homoeostasis and with the help of the biochemist James B Collip (1892–1965) successfully isolated insulin in 1922 (21, 22). Although all members of the team were listed as publication co-authors, the relationship between Banting and Best on one side and Macleod on the other rapidly deteriorated, the former group being of the opinion that their contributions in the discovery of insulin far outweighed those of Macleod. Much controversy emanated from the identification of insulin and, although a variety of versions regarding the saga exist, all concur that considerable acrimony was felt by all parties involved. The Nobel Prize committee further accentuated the tension by ignoring the contributions of Best and Collip with the result that Banting shared his Prize money with Best and Macleod provided half his award to Collip. The issue was further inflamed by the lack of recognition provided to the Romanian physiologist Nicolae C Paulescu (1869–1931) who had 8 months before Banting and Best's paper reported the discovery of a pancreas extract (named: ‘pancrein’), which lowered the blood glucose concentration (23).

After 1923, John Macleod further pursued his glucose homoeostasis interest at the Marine Biological Station in St Andrews, New Brunswick where he studied pancreatic insulin secretion in teleost fish. In 1928, he returned to Scotland, becoming Regius Professor of Physiology and subsequently Dean of the University of Aberdeen Medical Faculty, dying on 16 March 1935, aged 58 years (22, 24, 25, 26, 27, 28).

## The Nobel Prize in Physiology or Medicine 1947

The Nobel Prize in Physiology or Medicine 1947 was awarded to Carl Ferdinand Cori and Gerty Theresa Cori Radnitz ‘for their discovery of the course of the catalytic conversion of glycogen’ and to Bernardo Alberto Houssay ‘for his discovery of the part played by the hormone of the anterior pituitary lobe in the metabolism of sugar’.

### Bernardo Houssay

Bernardo Alberto Houssay was born in Buenos Aires on 10 April 1887, the son of French immigrants to Argentina. Despite being only 14 years old, he was admitted to the Pharmacy School at the University of Buenos Aires in 1901 and at the age of 17, in 1904, he entered from Buenos Aires the Medical School of the University. In 1908, he became the assistant lecturer in Physiology and in 1911 completed his MD thesis on the physiological activities of pituitary extracts. Thereafter, he was appointed Professor of Physiology in the University's School of Veterinary Medicine and in 1913, became Chief Physician at the Alvear Hospital in Buenos Aires. In 1915, he became Chief of the Section of Experimental Pathology at the National Public Health Laboratories in Buenos Aires and, in 1919, Houssay was appointed to the Chair of Physiology at the University of Buenos Aires Medicine School. In 1920, he married the chemist Dr Maria Angelica Catan (1895–1962). They had three children.

During the political unrest between 1943 and 1955, Houssay was forced to relocate his research to the Instituto de Biología y Medicina Experimental. However, in 1955, he was reinstated at the University of Buenos Aires, where he remained until his death. From 1957, Houssay was the director of the National Scientific and Technical Research Council of Argentina and in July 1960, Houssay he was elected the president of the ‘First International Congress of Endocrinology’ in Copenhagen (Denmark).

Houssay's main contribution was on the experimental investigation of the role of the anterior pituitary in the metabolism of carbohydrates, particularly in diabetes mellitus. He demonstrated the diabetogenic effect of anterior pituitary extracts and showed that the severity of diabetes decreased after anterior hypophysectomy (29, 30). These discoveries were instrumental in initiating research into the mechanistic basis of hormonal feedback mechanisms. Bernardo Houssay was widely acclaimed as a scientist and his contributions led to the award of numerous prizes ranging from that of the National Academy of Sciences, Buenos Aires, in 1923 to the Dale Medal of the Society of Endocrinology (London) in 1960. He died at the age of 84, on 21 September 1971 (31, 32, 33, 34, 35, 36, 37).

## The Nobel Prize in Physiology or Medicine 1950

The Nobel Prize in Physiology or Medicine 1950 was awarded jointly to Edward Calvin Kendall, Tadeus Reichstein and Philip Showalter Hench ‘for their discoveries relating to the hormones of the adrenal cortex, their structure and biological effects’.

### Edward Kendall

Edward Calvin Kendall was born on 8 March 1886 in South Norwalk, Connecticut (USA), and attended Columbia University, New York, New Jersey (USA), earning a Bachelor of Science degree in 1908, an MSc degree in Chemistry in 1909 and a PhD in Chemistry in 1910. From 1911 to 1914, he was employed by Parke, Davis and Company in Detroit, Michigan (USA). In 1915, he published his work on thyroid hormone and in so doing reported the first isolation of thyroxin (38, 39). Using similar strategies, Kendall and co-workers subsequently isolated and crystallised glutathione. In 1914, he moved to St Luke's Hospital in New York where he continued his research for a year until becoming Head of the Biochemistry Section, later Director of the Division of Biochemistry and Professor of Physiological Chemistry in the Graduate School of the Mayo Foundation, Rochester, Minnesota. Kendall married Rebecca Kennedy (1892–1973) in 1915. They had four children.

Despite his original seminal work on thyroxine, Kendall was mostly recognised for the isolation, identification and purification of several adrenal steroids (40, 41, 42). One of these isolated steroids was designated ‘compound E’ by Kendall and subsequently became better known as ‘cortisone’ (40, 41, 42). In studies in collaboration with Philip S Hench, cortisone proved to be a very effective drug in the treatment of rheumatoid arthritis. After retirement in 1951, Edward Kendall (43) became a Visiting Professor at Princeton University in Princeton, New Jersey (USA), remaining in Princeton until his death on 4 May 1972 at the age of 86 years (44, 45).

### Tadeus Reichstein

Tadeus Reichstein was born on 20 July 1897 in Wloclawek (Poland) (German: Leslau) and spent his early years in Kiev (Ukraine). His early schooling was in Jena (Germany) and thereafter from 1916 to 1920 he was educated at the Federal Institute of Technology in Zurich (Switzerland), where he obtained his PhD in 1922 and acquired Swiss citizenship. In 1922 in Zurich, he began the analysis of the chemical compounds that provide coffee and chicory their distinctive aromas (46). In 1927, he married the Dutch Lady Henriëtte Louise Quarles van Ufford (1898–1993). They had one daughter. Two years after their marriage (1929), Reichstein was appointed lecturer in organic and pharmaceutical chemistry at the University of Basel (Switzerland) and in 1933, he synthesised vitamin C (ascorbic acid) using a specific chemical procedure which was subsequently referred to as the ‘Reichstein process’. In 1938, he became Professor in Pharmaceutical Chemistry and Director of the Pharmaceutical Institute in Basel (1938–1950) and, in 1946, was appointed to the Chair of Organic Chemistry. During this time, Tadeus Reichstein collaborated with Edward C Kendall and Philip S Hench in their cortisone experiments. From 1953 to 1954, he collaborated with James F Tait and Sylvia A S Simpson Tait (London, UK), Albert Wettstein and Robert Neher (Ciba Ltd., Basel, Switzerland), and Marius Tausk (Organon, Oss, The Netherlands) in the isolation and characterisation of aldosterone (47). In 1960, he became Director of the Institute of Organic Chemistry in Basel. Reichstein was the recipient of numerous awards including the Benoist Prize of 1947 and the Copley Medal in 1968. Tadeus Reichstein died on 1 August 1996 in Basel at the age of 99 years (44, 48, 49).

### Philip Hench

Philip Showalter Hench was born on 28 February 1896 in Pittsburgh, Pennsylvania (USA). He received his undergraduate education at Lafayette College in Easton, Pennsylvania. In 1920, he was awarded a MD degree from the University of Pittsburgh having initially studied in the Medical Corps of the United States Army and the Reserve Corps. In 1923, he became a Fellow and thereafter in 1926, Head of the Department of Rheumatic Diseases at the Mayo Clinic, Rochester, Minnesota (USA). Hench focused his interest on arthritis and was one of the early observers of the fact that rheumatoid arthritis followed a milder course during pregnancy and jaundice (50, 51) and concluded that this phenomenon was due to a specific chemical compound (which later became known as ‘steroid’). Hench married Mary Genevieve Kahler (1905–1982) in 1927. They had four children, one of whom Philip Kahler Hench (1930–2009) also became a rheumatologist. In 1928 and 1929, Hench furthered his studies at Freiburg University and at the von Müller Klinikum in Munich (Germany). Thereafter, he returned to the Mayo Clinic, where, in collaboration with Edward Kendall he undertook studies of the effect of compound E (cortisone) on patients afflicted by rheumatoid arthritis. These studies were initially hampered by difficulties in synthesis of the compound and thereafter by the advent of the Second World War. Between 1942 and 1946, Hench was a Lieutenant Colonel in the Medical Corps of the US army, retiring with the rank of Colonel. In 1947, he was appointed Professor of Medicine at the Mayo Clinic and restarted his studies with cortisone in the treatment of rheumatoid arthritis, which had been postponed by the war (52, 53). Hench was awarded many honours including the Heberdeen Medal (1942), the Lasker Award (1949) and the Passano Foundation Award (1950), as well as numerous honorary doctorates. He is also remembered for his witty speech at the banquet ceremony during the Nobel Prize ceremony where he remarked of his co-winners Reichstein and Kendall: ‘Perhaps the ratio of one physician to two chemists is symbolic, since medicine is so firmly linked to chemistry by a double bond’. His penchant for wit and diverse interests is also reflected in his important collection of original documents (Philip S Hench Walter Reed Yellow Fever Collection) pertinent to the history of the treatment of Yellow Fever, an important issue in American medical history of his time. Philip Hench died of pneumonia while on vacation in Ocho Rios, Jamaica, on 30 March 1965 at the age of 69 years (44, 54, 55, 56, 57, 58, 59, 60).

## The Nobel Prize in Physiology or Medicine 1971

The Nobel Prize in Physiology or Medicine 1971 was awarded to Earl Wilbur Sutherland, Jr ‘for his discoveries concerning the mechanisms of the action of hormones’.

### Earl Sutherland, Jr

Earl Wilbur Sutherland, Jr was born on 19 November 1915 in Burlingame, Kansas (USA), and, in 1933, enrolled in Washburn College, in Topeka, Kansas (USA). He graduated with a Bachelor of Science degree in 1937. In 1942, he was awarded a doctorate in medicine from the Washington University School of Medicine in St Louis, Missouri (USA), having been mentored as a student by Carl F Cori. Carl Cori and his wife Gerty T Cori Radnitz would jointly receive the Nobel Prize in Physiology or Medicine in 1947, ‘for their discovery of the course of the catalytic conversion of glycogen’. Working in their laboratory, Sutherland studied the effects of adrenaline and glucagon on the conversion of glycogen to glucose before becoming an intern in 1942 at Barnes Hospital, St Louis, Missouri (USA). In 1937, he married Mildred Rice. They had three children. Having received an MD degree in 1942, Sutherland became a battalion surgeon and staff physician in a military hospital in Germany (1942–1945), before returning to the Cori Laboratory at Washington University School of Medicine after the end of the Second World War. His initial appointment was as Instructor in Pharmacology from 1945, followed by Instructor in Biochemistry from 1946 to 1950. In 1950, he was promoted to Assistant Professor in Biochemistry and became Associate Professor in 1952. In 1953, he was appointed Professor of Pharmacology and Chairman of the Department of Pharmacology at the Case Western Reserve University in Cleveland, Ohio (USA), and initiated his lifelong collaboration with his research partner and Professor of Pharmacology, Theodore W Rall. Sutherland and Rall thereafter investigated the mechanisms of hormone action at the molecular level, culminating in the discovery of cyclic AMP and the identification of its role as a secondary messenger (61). In 1962, Sutherland divorced and moved in 1963 to Nashville, Tennessee (USA), becoming Professor of Physiology at Vanderbilt University School of Medicine. In the same year, he married Dr Claudia Sebeste Smith, the Assistant Dean at the University. After a decade at Vanderbilt University, Sutherland in 1973, moved to the Leonard M Miller School of Medicine in Miami, Florida (USA), to become Professor of Biochemistry. Earl Sutherland was awarded numerous honours including the Albert Lasker Award (1970) and the National Medal of Science (1973). On 9 March 1974, he died at the age of 58 of surgical complications for the management of oesophageal haemorrhage from portal hypertension (61, 62, 63, 64, 65, 66).

## The Nobel Prize in Physiology or Medicine 1977

The Nobel Prize in Physiology or Medicine 1977 was divided between Rosalyn Yalow ‘for the development of RIAs of peptide hormones’ and the other half jointly to Roger Guillemin and Andrew Victor Schally ‘for their discoveries concerning the peptide hormone production of the brain’.

### Rosalyn Yalow

Rosalyn Sussman was born on 19 June 1921 in New York, New Jersey (USA), and attended Walton High School before becoming a secretary for Rudolph Schoenheimer, a leading biochemist at Columbia University's College of Physicians and Surgeons. Thereafter, she became the secretary to Michael Heidelberger, immunologist at Columbia University, who hired her as she studied stenography. Sussman subsequently graduated from Hunter College (New York) in January 1941 (3) and moved as the only female teaching assistant in physics to the College of Engineering of the University of Illinois at Urbana–Champaign. In 1943, she married Aaron Yalow (1920–1992). They had two children. In 1945, Rosalyn Yalow earned her PhD in nuclear physics and acquired a position as the only woman assistant engineer at the Federal Telecommunications Laboratory in New York. In 1946, she returned to Hunter College to teach physics to returning war veterans, maintaining this responsibility until 1950. In 1948, Yalow joined the Bronx Veterans Administration Hospital (VAMC), New York, as a part-time consultant to assist Bernard Roswit, Chief of the Radiotherapy Department, to establish a radioisotope service and initiated research projects with him. In the Bronx VAMC, she met the internist Dr Salomon A Berson (1918–1972), who became her professional collaborator for the next 22 years. The classical basis of radioimmunoassay (RIA) was established by Berson and Yalow leading to the development of the insulin RIA in 1959 (67). Thereafter, the RIA technique became developed to measure numerous hormones or other substances in body fluids. Despite its immense commercial potential, Yalow and Berson declined to patent their methodology. In 1968, when Salomon Berson was named Murray M Rosenberg Professor and Chair of Medicine at Mount Sinai School of Medicine of the City University of New York, New Jersey (USA), Yalow was appointed Research Professor in the Department of Medicine and subsequently, after Berson's death, the Solomon Berson Distinguished Professor at Large. Despite his appointment to Mount Sinai School of Medicine, Berson maintained his laboratory investigative centre at the Bronx VAMC as Yalow had been vehemently opposed to his accepting the chair of Medicine at Mount Sinai School of Medicine. In 1975, Yalow and Berson (posthumously) were awarded the American Medical Association (AMA) Scientific Achievement Award and, in 1976, Yalow became the first female recipient of the Albert Lasker Award for Basic Medical Research. In 1978 she was elected a Fellow of the American Academy of Arts and Sciences and received the National Medal of Science in 1988. Rosalyn Yalow died on 30 May 2011, aged 89, in New York (68, 69, 70, 71).

### Roger Guillemin

Roger Charles Louis Guillemin was born on 11 January 1924 in Dijon (Bourgogne, France), undertook his undergraduate work at the University of Burgundy and received an MD degree from the Faculté de Médecine of Lyon in 1949. During the Second World War, he was active in the French resistance (for this heroism he was decorated with the ‘Ordre national de la Légion d'honneur’ in 1973). In 1951, he nearly died of tuberculous meningitis, but recovered and married his nurse, Lucienne Jeanne Billard. Later she became a famous professional harpsichord player. They became American citizens in 1965 and they had six children.

In 1949, Guillemin began work with Hans H B Selye at the Institute of Experimental Medicine and Surgery at the McGill University in Montréal (Canada) receiving a PhD in 1953 and then moving to Baylor College of Medicine at Houston, Texas (USA). In 1970, he helped establish the Salk Institute in La Jolla, California (USA) and continued his scientific work there at until retirement in 1989.

His research team focused on unravelling the hypothalamic biochemical regulation of anterior pituitary function and secretion. Independent of, and also in competition with, the research group of Andrew Schally, Roger Guillemin and his co-workers discovered the following hormones: thyrotrophin-releasing hormone (TRH), growth hormone-releasing hormone (GHRH) and somatostatin (72, 73, 74, 75, 76, 77). In addition to hormonal structure function investigation, Guillemin addressed the subjects of activins, inhibins and fibroblast growth factor (FGF). Guillemin was awarded numerous honours and prizes including the National Academy of Sciences (1974), the Gairdner Foundation International Award (1974), the Albert Lasker Award (1975), the American Academy of Arts and Sciences Prize (1976), the Dickson Prize in Medicine (1976), the Passano Award in Medical Sciences (1976) and the National Medal of Science (1976). After his retirement, Guillemin became an artist creating abstract impressionist art using a Macintosh computer to create images on paper or canvas.

### Andrew Schally

Andrzej Viktor ‘Andrew’ Schally was born on 30 November 1926 in Wilno, Poland (now Vilnius, Lithuania), and survived the Polish Holocaust when his family fled to neutral Romania. In 1945, he moved to the United Kingdom, where he finished high school. In 1950, he joined the National Institute of Medical Research Mill Hill in London (UK), moving in 1952 to McGill University in Montreal (Canada). In 1955, together with Murray Saffran, he demonstrated the presence of corticotrophin-releasing hormone in the hypothalamus and posterior pituitary (78) and received his doctorate in endocrinology in 1957. Upon award of his PhD, Schally departed to the United States becoming Assistant Professor of Physiology and Senior Research Fellow of the US Public Health Service at Baylor University College of Medicine in Houston, Texas (1957–1962). At this time, Schally still collaborated closely with Roger Guillemin although subsequently they became competitors. In 1962, Schally acquired US citizenship and accepted the position of Assistant Professor of Medicine at Tulane University Medical School in New Orleans, Louisiana (USA), focusing his research primarily on neuropeptides. His research group studied TRH (79), luteinising hormone-releasing hormone (LHRH), GHRH, bombesin/gastrin-releasing peptide (80) and somatostatin (80, 81) as well as developing LHRH analogues for the treatment of prostate cancer (82, 83). In addition, Schally developed therapeutic strategies including cytotoxic analogues of LHRH, GHRH (84), bombesin and somatostatin (80, 85) with an ultimate goal of producing therapies for various solid tumours and non-Hodgkin lymphoma. Schally's scientific output before his receipt of the Nobel Prize has been only matched by his productivity thereafter. Following the devastating effects of hurricane Katrina in 2005, Schally moved to Miami, Florida (USA), becoming Chief of the new Endocrine, Polypeptide and Cancer Institute at the VA Medical Center in Miami and Distinguished Medical Research Scientist of the Veterans Affairs Department, USA. Currently, he is the Distinguished Leonard M Miller Professor of Pathology and also Professor of Medicine in the Division of Hematology/Oncology at the University of Miami, Miller School of Medicine. Schally was married twice. First to Margaret Rachel White with whom he had two children and, in 1976, he married the Brazilian endocrinologist Ana Maria de Medeiros-Comaru with whom he published many papers (81). Andrew Schally has won many prizes and was awarded many honorary doctorates an MD *honoris causa* from the Jagiellonian University in Cracow, Poland.

## Other Nobel Prizes

Other Nobel Prizes in Physiology or Medicine, or in Chemistry for research into areas closely related to endocrinology were as follows:

## The Nobel Prize in Chemistry 1939

The Nobel Prize in Chemistry 1939 was divided equally between *Adolf (Friedrich Johann) Butenandt* (1903–1995) ‘for his work on sex hormones’ and Leopold Ružička (1887–1976) ‘for his work on polymethylenes and higher terpenes’.

## The Nobel Prize in Chemistry 1955

The Nobel Prize in Chemistry 1955 was awarded to *Vincent du Vigneaud* (1901–1978) ‘for his work on biochemically important sulphur compounds, especially for the first synthesis of a polypeptide hormone’.

## The Nobel Prize in Chemistry 1958

The Nobel Prize in Chemistry 1958 was awarded to *Frederick Sanger* (1918–2013) ‘for his work on the structure of proteins, especially that of insulin’.

## The Nobel Prize in Physiology or Medicine 1964

The Nobel Prize in Physiology or Medicine 1964 was awarded jointly to *Konrad (Emil) Bloch* (1912–2000) and *Feodor (Felix Konrad) Lynen* (1911–1979) ‘for their discoveries concerning the mechanism and regulation of the cholesterol and fatty acid metabolism’.

## The Nobel Prize in Physiology or Medicine 1966

The Nobel Prize in Physiology or Medicine 1966 was divided equally between (Francis) Peyton Rous (1879–1970) ‘for his discovery of tumour-inducing viruses’ and *Charles (Brenton) Huggins* (1901–1997) ‘for his discoveries concerning hormonal treatment of prostatic cancer’.

## The Nobel Prize in Physiology or Medicine 1970

The Nobel Prize in Physiology or Medicine 1970 was awarded jointly to *Sir Bernard Katz* (1911–2003), *Ulf (Svante) von Euler* (1905–1983) and *Julius Axelrod* (1912–2004) ‘for their discoveries concerning the humoral transmitters in the nerve terminals and the mechanism for their storage, release and inactivation’.

## The Nobel Prize in Physiology or Medicine 1982

The Nobel Prize in Physiology or Medicine 1982 was awarded jointly to *Sune (Karl) Bergström* (1916–2004), *Bengt (Ingemar) Samuelsson* (1934) and *John (Robert) Vane* (1927–2004) ‘for their discoveries concerning prostaglandins and related biologically active substances’.

## The Nobel Prize in Physiology or Medicine 1985

The Nobel Prize in Physiology or Medicine 1985 was awarded jointly to *Michael (Stuart) Brown* (1941) and *Joseph (Leonard) Goldstein* (1940) ‘for their discoveries concerning the regulation of cholesterol metabolism’.

## The Nobel Prize in Physiology or Medicine 1986

The Nobel Prize in Physiology or Medicine 1986 was awarded jointly to *Stanley Cohen* (1922) and *Rita LeviMontalcini* (1909–2012) ‘for their discoveries of growth factors’.

## The Nobel Prize in Physiology or Medicine 1991

The Nobel Prize in Physiology or Medicine 1991 was awarded jointly to *Erwin Neher* (1944) and *Bert Sakmann* (1941) ‘for their discoveries concerning the function of single ion channels in cells’.

## The Nobel Prize in Physiology or Medicine 1994

The Nobel Prize in Physiology or Medicine 1994 was awarded jointly to *Alfred (Goodman) Gilman* (1941) and *Martin Rodbell* (1925–1998) ‘for their discovery of G-proteins and the role of these proteins in signal transduction in cells’.

## The Nobel Prize in Physiology or Medicine 2000

The Nobel Prize in Physiology or Medicine 2000 was awarded jointly to *Arvid Carlsson* (1923), Paul Greengard (1925) and Eric (Richard) Kandel (1929) ‘for their discoveries concerning signal transduction in the nervous system’. Arvid Carlsson discovered that dopamine is a transmitter in the mammalian brain.

## The Nobel Prize in Physiology or Medicine 2010

The Nobel Prize in Physiology or Medicine 2010 was awarded to *Sir Robert (Geoffrey) Edwards* (1925–2013) ‘for the development of IVF’.

## Figures and Tables

**Table 1:** Nobel Prize winners in endocrinology.

**Year**	**Recipients**	**Award**
2010 (PM)	Robert G Edwards (UK)	IVF
2000 (PM)	Arvid Carlsson	Dopamine
1994 (PM)	Alfred G Gillman, Martin Rodbell	G-proteins and their function
1986 (PM)	Stanley Cohen, Rita Levi Montalcini	Growth factors
1985 (PM)	Michael S Brown, Joseph L Goldstein	Cholesterol metabolism
1982 (PM)	Sune K Bergström, Bengt I Samuelsson, John R Vane	Prostaglandins
1977 (PM)	Roger Guillemin, Andrew V Schally	Hypothalamic peptides
1977 (PM)	Rosalyn Yalow	RIA
1971 (PM)	Earl W Sutherland, Jr	Mechanism of action of hormones
1970 (PM)	Bernard Katz, Ulf S von Euler, Julius Axelrod	Acetylcholine and catecholamines
1966 (PM)	Charles B Huggins	Hormonal treatment prostatic cancer
1964 (PM)	Konrad E Bloch, Feodor F K Lynen	Cholesterol and fatty acid metabolism
1958 (Ch)	Frederick Sanger	Structure of insulin
1955 (Ch)	Vincent du Vigneaud	Synthesis of oxytocin
1950 (PM)	Edward C Kendall, Tadeus Reichstein, Philip S Hench	Adrenocortical hormones
1947 (PM)	Carl F Cori and Gerty T Cori Radnitz	Catalytic conversion of glycogen
1947 (PM)	Bernardo A Houssay	Anterior pituitary and carbohydrate metabolism
1939 (Ch)	Adolf F J Butenandt	Sex hormones
1923 (PM)	Frederick G Banting, John J R Macleod	Discovery of insulin
1909 (PM)	E Theodor Kocher	Physiology, pathology and surgery of the thyroid gland
1901 (Ph)	Wilhelm C Röntgen	X-rays

PM, physiology or medicine; Ph, physics; Ch, chemistry.
